# Persistence of an Infectious Form of SARS-CoV-2 After Protease Inhibitor Treatment of Permissive Cells In Vitro

**DOI:** 10.1093/infdis/jiae385

**Published:** 2024-08-12

**Authors:** Manoj S Nair, Maria I Luck, Yaoxing Huang, Yosef Sabo, David D Ho

**Affiliations:** Aaron Diamond AIDS Research Center, Columbia University Vagelos College of Physicians and Surgeons, New York, New York, USA; Division of Infectious Diseases, Department of Medicine, Columbia University Vagelos College of Physicians and Surgeons, New York, New York, USA; Aaron Diamond AIDS Research Center, Columbia University Vagelos College of Physicians and Surgeons, New York, New York, USA; Division of Infectious Diseases, Department of Medicine, Columbia University Vagelos College of Physicians and Surgeons, New York, New York, USA; Aaron Diamond AIDS Research Center, Columbia University Vagelos College of Physicians and Surgeons, New York, New York, USA; Division of Infectious Diseases, Department of Medicine, Columbia University Vagelos College of Physicians and Surgeons, New York, New York, USA; Aaron Diamond AIDS Research Center, Columbia University Vagelos College of Physicians and Surgeons, New York, New York, USA; Division of Infectious Diseases, Department of Medicine, Columbia University Vagelos College of Physicians and Surgeons, New York, New York, USA; Aaron Diamond AIDS Research Center, Columbia University Vagelos College of Physicians and Surgeons, New York, New York, USA; Division of Infectious Diseases, Department of Medicine, Columbia University Vagelos College of Physicians and Surgeons, New York, New York, USA; Department of Microbiology and Immunology, Columbia University Vagelos College of Physicians and Surgeons, New York, New York, USA

**Keywords:** SARS-CoV-2, protease inhibitor, nirmatrelvir, rebound, virus persistence

## Abstract

Reports have described severe acute respiratory syndrome coronavirus 2 (SARS-CoV-2) rebound in coronavirus disease 2019 (COVID-19) patients treated with nirmatrelvir, a 3CL protease inhibitor. The cause remains a mystery, although drug resistance, reinfection, and lack of adequate immune responses have been excluded. We now present virologic findings that provide a clue to the cause of viral rebound, which occurs in approximately 20% of the treated cases. Persistence of infectious SARS-CoV-2 was experimentally documented in vitro after treatment with nirmatrelvir or another 3CL protease inhibitor, but not with a polymerase inhibitor, remdesivir. This infectious form decayed slowly with a half-life of approximately 1 day, suggesting that its persistence could outlive the treatment course to reignite SARS-CoV-2 infection as the drug is eliminated. Notably, extending nirmatrelvir treatment beyond 8 days abolished viral rebound in vitro. Our findings point in a particular direction for future investigation of virus persistence and offer a specific treatment recommendation that should be tested clinically.

## Teaser

SARS-CoV-2 persists in the current regimen of Paxlovid with lingering infectivity that rebounds after the course. The half-life of this infectious form is about a day and is eliminated under longer treatment course.

Paxlovid, a Food and Drug Administration approved drug to treat symptomatic severe acute respiratory syndrome coronavirus 2 (SARS-CoV-2) infection in elderly and high-risk individuals [1], is an oral combination of nirmatrelvir, an inhibitor of the main protease (3CL) of SARS-CoV-2, and ritonavir, a CY3PA inhibitor that boosts the plasma concentration of nirmatrelvir [2]. Other protease inhibitors approved for clinical use include ensitrelvir (also known as S-217622) [3] in Japan, and simnotrelvir (also known as SIM0417) [4] in China.

Numerous coronavirus disease 2019 (COVID-19) patients receiving the recommended 5-day course of Paxlovid (300 mg nirmatrelvir/100 mg ritonavir every 12 hours) became symptomatically better and virus negative only to have a rebound of detectable SARS-CoV-2 again 2–8 days later [5]. Many of these cases had a recurrence of symptoms, albeit mild. The Centers for Disease Control and Prevention issued a health advisory because of concerns for forward transmission of the virus during its recrudescence [6], even as another case series was reported [7]. Several retrospective studies suggested the prevalence of “Paxlovid rebound” was low at approximately 1%–2% [8–10], but a couple of prospective studies have shown the rates to be as high as 20%–27% [11, 12], as many medical practitioners have noted anecdotally. These reports showed that the low prevalence found in prior retrospective studies was largely due to inadequate posttreatment sampling to detect the virus. Additionally, several studies suggested viral relapse was common in untreated patients [13, 14], but these were descriptions of viral “blips” that were not sustained. Moreover, the infrequency (0.7%) of SARS-CoV-2 rebound in the absence of therapy is well documented in a large study of infected individuals (n = 999) who were closely monitored [15]. An explanation for the viral relapse remained elusive [16], although viral resistance to nirmatrelvir, inadequate adaptive immunity, and reinfection were ruled out [5, 7, 11, 17, 18]. In this study, we probed the underlying cause of the viral rebound by assessing the persistence of infectious SARS-CoV-2 in several permissive cell lines after treatment with high doses of nirmatrelvir or ensitrelvir in vitro.

## METHODS

### Cell Lines and Virus Strains

Huh7 cells and A549 cells engineered to express human ACE2 protein and *Cercopithecus aethiops* kidney epithelial cells TMPRSS2 protein were used in the current study. Details on the expression of the proteins and their culturing are provided in Supplementary Methods. Viruses used in the study are detailed in Supplementary Methods.

### Drugs

Nirmatrelvir (PF-07321332), ensitrelvir (S-217622), GC-376, and remdesivir were purchased from Medkoo Biosciences. All drugs were >96% pure and dissolved in dimethyl sulfoxide (DMSO) prior to using in dose-response and infectivity experiments. Drugs were diluted in culture media to reduce effective concentrations of DMSO to <0.2%.

### Determination of Minimal Cell Number for Recovering Infectivity

Virus end point titrations were performed to determine the efficacy of protease and polymerase inhibitors at doses 10-fold or higher than their 99% inhibitory concentrations (IC_99_) in 2 or more cell lines. Three formats were used to determine the infectivity levels in vitro, referred to as pretreatment, concurrent treatment, or posttreatment.

For pretreatment formats, nirmatrelvir, ensitrelvir, or remdesivir was added to a monolayer of Huh7-ACE2 cells at doses 10-fold of their IC_99_ for 6 hours prior to infecting them with infectious-clone derived (ic)-SARS-CoV-2/WA1-mNG at 0.5 multiplicity of infection (MOI). Cells were incubated with drugs and/or virus at 37°C/5% CO_2_ for 24, 48, or 72 hours. At each time point, media containing drug and/or free virions were removed, and cells were washed 3 times with phosphate-buffered saline. After washes, the cells were trypsinized with 0.25% trypsin-EDTA (37°C/5% CO_2_ for 3 minutes) and collected in Eagle's minimum essential medium (EMEM) with 10% fetal calf serum. Cells were centrifuged and washed again before the cell pellet was resuspended into 200–250 µL EMEM and counted using a Bio-Rad TC10 automated cell counter. Counted cells were serially diluted 3-fold in EMEM to set up an end point titration in 96-well plates starting at 22 500 cells/well and 100 µL of each dilution was overlaid on Vero cells expressing ACE2 and TMPRSS2 (Vero-TMPRSS2), which are extremely sensitive to virus replication and exhibit prominent cytopathic effects (CPE). The dilutions were incubated at 37°C/5% CO_2_ for 72 hours prior to determining the viral end point titer from each of the treated concentrations of the extract. Three replicates of titration were set up for each drug for each experiment. Each experiment was performed in triplicates and the minimal cell number from an experiment is plotted for each time point at which the drug was removed from the medium.

For the concurrent treatment format of experiments, we used 3 different cell lines for the study. Huh7-ACE2 and A549-ACE2 human cell lines of liver or lung origin were infected with ic-SARS-CoV-2/WA1-mNG at 0.5 MOI for 10 minutes prior to adding nirmatrelvir, ensitrelvir, GC376, or remdesivir at concentrations of 10-fold or higher than their IC_99_. A more rigorous time course was used for this study by performing removal of drug at 10, 24, 48, 72, and 96 hours postinfection. The drug removal was performed similarly to the pretreatment format and 3-fold end point dilutions starting at 22 500 cells/well were placed into a monolayer of Vero-TMPRSS2 cells. For infection with Omicron BA.1.1 isolate, Vero-TMPRSS2 cells were infected at 0.5 MOI for 10 minutes prior to drug treatment as above. Following drug removal, the end point titration was performed using 3-fold dilutions of the collected cells onto a new monolayer of Vero-TMPRSS2 cells. The diluted cells were incubated at 37°C/5% CO_2_ for 72 hours prior to determining the viral end point titer from each of the treated concentrations of the extract. Three replicates of titration were set up for each drug for each experiment. Each experiment was performed in triplicates, and the minimal cell number needed to recover infectious virus from an experiment is plotted for each time point when the drug was removed from the medium.

For the postinfection treatment format, Huh7-ACE2 cells were infected with ic-SARS-CoV-2/WA1-mNG at 0.5 MOI for 6 hours in 37°C/5% CO_2_. Thereafter, the infection medium was replaced with a medium containing nirmatrelvir or remdesivir at a concentration 10-fold higher than its 99% inhibitory dose (IC_99_) in the cells. Cells were incubated at 37°C/5% CO_2_ for 24, 48, or 72 hours, after which the cells were washed to remove the drug and free virion, trypsinized, washed again, and counted. The collected cells were then diluted 3-fold starting at 2500 cells/well and overlaid onto a fresh monolayer of Vero-TMPRSS2 cells. Three replicates of each titration were set up for each drug, and each experiment was performed in triplicate. Minimal cell numbers needed to yield infectious virus obtained from the experiment were plotted for each time point when the drug was removed.

For the long-term postinfection treatment study, Huh7-ACE2 cells were infected with ic-SARS-CoV-2/WA1-mNG at 0.5 MOI for 6 hours in 37°C/5% CO_2_. Details of this method are described in Supplementary Methods.

### Single-Molecule RNA FISH and Immunofluorescence

Simultaneous detection of intracellular SARS-CoV-2 viral genomic RNA and nucleocapsid protein using immunofluorescence combined single-molecule RNA fluorescence in situ hybridization (FISH) was performed as described by Kochan et al [19] with minor modifications, as described in the Supplementary Methods.

### Microscopy

Images were acquired using motorized spinning-disc confocal microscope (Yokogawa CSU-X1 A1 confocal head and Zeiss Axio Observer Z1 microscope) using × 63 oil (1.4 NA) and × 20 dry (0.8 NA) objectives. Three-dimensional z stacks were acquired for cells displaying nucleocapsid signal and images were analyzed with SlideBook software (Intelligent Imaging Innovations; SlideBook version 6.0.24 [40297]). For figures, 2-dimensional projection images of the maximum pixel intensity over the z axis were generated.

### Viral Genomic RNA and Nucleocapsid Quantification

Intensity measurement of viral nucleocapsid or viral genomic RNA (gRNA) were achieved by intensity-based segmentation using manually defined thresholding in SlideBook Software. Signal intensity distribution was plotted as a violin plot and statistical significance was determined by 1-tailed Student *t* test in GraphPad Prism version 9.4.

### Quantitation and Statistical Analysis

Details of quantitative analysis to measure decay half-life and statistical analysis are reported in Supplementary Methods.

## RESULTS

To ensure maximum inhibition of SARS-CoV-2, we first determined the inhibitory potency of nirmatrelvir and ensitrelvir (3CL protease inhibitors), and remdesivir (a polymerase inhibitor control) in 2 different permissive cell lines, Huh7-ACE2 and A549-ACE2. All the drugs showed robust virus inhibition in all the cell lines, as indicated by their 50% and 99% inhibitory concentrations (IC_50_ and IC_99_) (Supplementary Figure 1*A*). All drugs were nontoxic at these doses in the cell lines and therefore suitable for measuring the effects on persistence (Supplementary Figure 1*D*)

**Figure 1.**
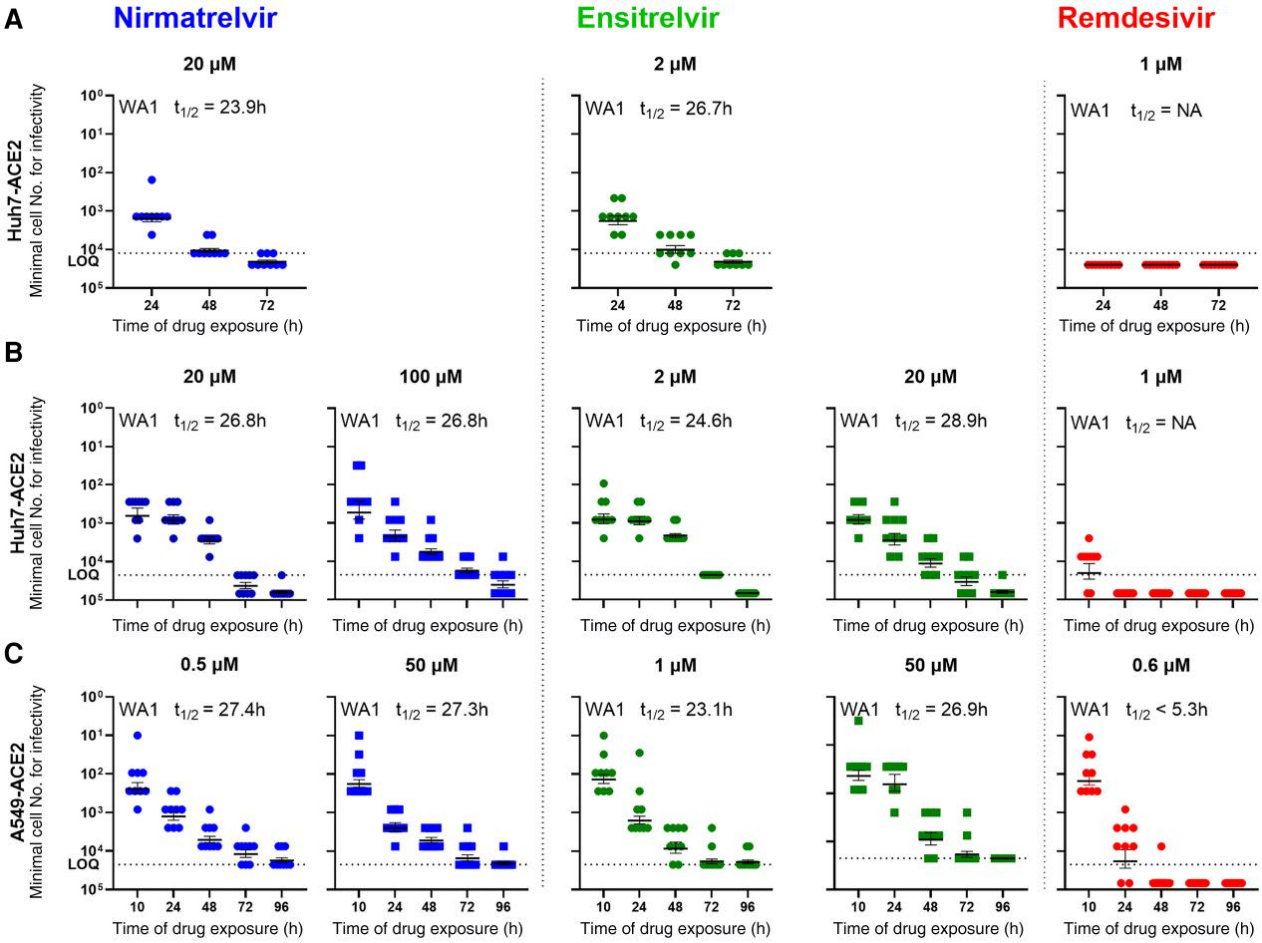
SARS-CoV-2 persistence in cells pretreated or concurrently treated with protease or polymerase inhibitor at doses 10 × IC_99_ or higher. Minimum number of cells retaining infectious virus present after removal of drug treatment at indicated time points are shown in each panel. *A*, Infectivity decay in Huh7-ACE2 cells when treated 6 hours prior to infection at approximately 10 × IC_99_. *B*, Infectivity decay in Huh7-ACE2 cells when treated concurrently (within 10 minutes of infection) with ≥10 × IC_99_ (1.8 µM nirmatrelvir, 180 nM ensitrelvir, and 80 nM Remdesivir). *C*, Infectivity decay in A549-ACE2 cells when treated concurrently with ≥10 × IC_99_ (35 nM nirmatrelvir, 90 nM ensitrelvir, and 60 nM Remdesivir). Dotted lines indicate the upper limit of input cells (maximum cell number from which end point titration was performed at 3-fold dilutions) for the assay. Half-life of the decay of the infectious form of the virus after treatment with the drug at specified concentrations is shown. Results for nirmatrelvir are shown in blue, ensitrelvir in green, and remdesivir in red. Error bars in each graph represent the mean ± SEM of the minimum number of cells for infectivity. Abbreviations: IC_99_, 99% inhibitory concentration; NA, not applicable; SARS-CoV-2, severe acute respiratory syndrome coronavirus 2; t_1/2_, half-life.

We next examined the persistence of infectious virus in Huh7-ACE2 for 3 consecutive days after treatment with each drug (nirmatrelvir, ensitrelvir, or remdesivir) at 10 × IC_99_ 6 hours prior to inoculation with SARS-CoV-2/USA/WA1 bearing the mNeonGreen reporter gene (SCoV-2/mNG) [20] at 0.5 MOI. At 24, 48, and 72 hours postinfection, batches of cells were washed, counted, and then subjected to a serial 3-fold titration for infectious virus starting with 12 500 cells per well into Vero-TMPRSS2 indicator cells free of drug. End point titers of infectious forms of SARS-CoV-2 showed an expected time-dependent decline after nirmatrelvir or ensitrelvir treatment (Figure 1*A*). However, infectious virus was detectable at all time points, at least in some of the wells. Using linear regression analysis of data, the decay half-lives of the infectivity were calculated to be 23.9 hours for nirmatrelvir and 26.7 hours for ensitrelvir. In contrast, remdesivir-treated cells had no measurable infectivity at all time points assessed (Figure 1*A*). These findings suggested that while nirmatrelvir or ensitrelvir could block the main viral protease [2, 21], a replication-competent form of the virus can persist intracellularly long enough to reinitiate infection once the drug is removed.

Next, we repeated a similar experiment adding additional time points as well as higher concentrations of protease inhibitors. Huh7-ACE2 cells were infected with SCoV-2/mNG at 0.5 MOI and concurrently treated with nirmatrelvir, ensitrelvir, or remdesivir at concentrations of 10 × IC_99_ or higher. As above, batches of cells were serially washed and harvested to measure the number of cells required to yield infectious virus. Cells treated with either nirmatrelvir or ensitrelvir retained an infectious form of SARS-CoV-2 at the first 3 time points (10, 24, and 48 hours) (Figure 1*B*). By 72 hours, only 10%–30% of replicates yielded detectable infectious virus from cells treated with either of the protease inhibitors. Importantly, even at 96 hours after nirmatrelvir treatment, a minor proportion of replicates (1/9) yielded infectious virus. As before, the rates of infectivity decay were calculated to be 26.8 hours and 24.6–28.9 hours for nirmatrelvir and ensitrelvir, respectively (Figure 1*B*). Similarly, the infectivity decay in cells treated with remdesivir was substantially faster, with no infectious virus detected by 24 hours.

To exclude cell type-specific effects, we performed a similar experiment using the human lung carcinoma-derived alveolar epithelial cell line (A549-ACE2). Both nirmatrelvir and ensitrelvir were more potent in A549-ACE2 cells than in Huh7-ACE2 cells (Supplementary Figure 1*B*); however, the infectivity decay results obtained in A549-ACE2 cells (Figure 1*C*) were comparable to those in Huh7-ACE2 cells. The calculated infectivity decay half-lives were 27.3–27.4 hours for nirmatrelvir, 23.1–26.9 hours for ensitrelvir, and <5.3 hours for remdesivir.

**Figure 2.**
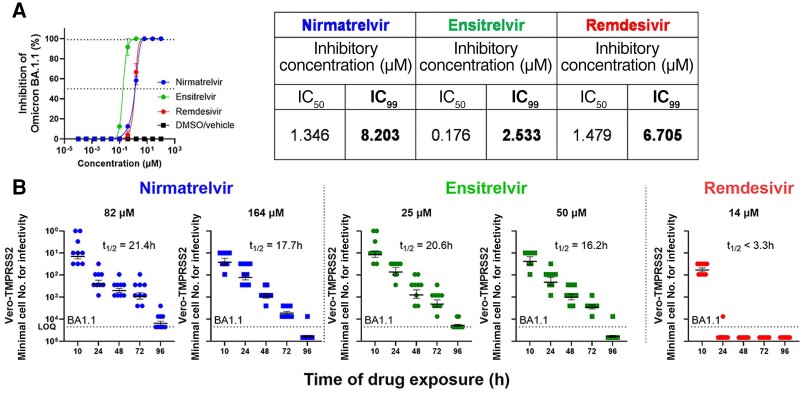
Persistence of SARS-CoV-2 Omicron BA.1.1 concurrently treated with protease inhibitors at doses 10 × IC_99_ or higher. Minimum numbers of cells retaining infectious virus present after removal of drug treatment at indicated time points are shown. *A*, Dose titration of drugs/DMSO vehicle in Vero-TMPRSS2 cells infected with Omicron BA1.1 virus. All viruses were used at MOI 0.5 to obtain the IC_50_ and IC_99_ of inhibition of the proteases and polymerase inhibitor. The calculated IC_50_ and IC_99_ for each drug tested are shown. *B*, Minimum number of cells retaining infectious virus present after removal of drug treatment at indicated time points are shown. The dotted line indicates the upper limit of input cells (maximum cell number from which end point titration was performed at 3-fold dilutions) for the assay. The half-life of the decay of infectious form of the virus after treatment with the drug at the specified concentrations is shown. Results for nirmatrelvir are shown in blue, ensitrelvir in green, and remdesivir in red. Error bars in each graph represent the mean ± SEM of the minimum cell number for infectivity. Abbreviations: DMSO, dimethyl sulfoxide; IC_99_, 99% inhibitory concentration; IC_50_, 50% inhibitory concentration; SARS-CoV-2, severe acute respiratory syndrome coronavirus 2; t_1/2_, half-life.

Paxlovid rebound was first reported [5] when the Omicron variant of SARS-CoV-2 was most prevalent. We therefore conducted a similar experiment using the Omicron BA.1.1 virus and the susceptible Vero-TMPRSS2 cell line after dose titration of the same drugs against the variant (Figure 2*A*). The lack of reporter gene and the enhanced permissiveness of the isolate in Vero-TMPRSS2 cells were reasons to choose these cells for treatment and end point study. Cells were concurrently inoculated with the virus (0.5 MOI) and treated with 10 × IC_99_ or higher concentrations of nirmatrelvir or ensitrelvir and 10 × IC_50_ concentration of remdesivir. Similar patterns of infectivity decay were observed once more (Figure 2*B*). Calculated decay rates were slightly faster in this experiment, with half-lives of 17.7–21.4 hours for nirmatrelvir and 16.2–20.6 hours for ensitrelvir. The infectivity decay for remdesivir was again substantially faster, with half-life of <3.3 hours and no detectable infectious virus after 24 hours of treatment.

We then tested a third protease inhibitor, GC-376, that was reported to inhibit SARS-CoV-2 in vitro [22], because simnotrelvir [23] was not available in the United States. Dose titration on this investigational compound was again assessed (Supplementary Figure 2) in Huh7-ACE2 and Vero-TMPRSS2 cells. Both cell lines were inoculated with the virus and treated with nirmatrelvir or ensitrelvir at concentrations of approximately 10 × IC_99_, as previously described. The infectivity decay in both cell lines resembled those of nirmatrelvir and ensitrelvir shown in Figure 1, with calculated half-lives of 28.1 hour for WA1 strain in Huh7-ACE2 cells and 20.3 hours for the Omicron BA.1.1 strain in Vero-TMPRSS cells (Supplementary Figure 2). Therefore, treatment with all 3 protease inhibitors in vitro led to similar persistence of an infectious form of SARS-CoV-2 that decayed slowly with a half-life of approximately 1 day for the WA1 strain and slightly shorter for Omicron BA.1 or BA.1.1. In contrast, treatment with a polymerase inhibitor resulted in a rapid loss of infectious SARS-CoV-2 in vitro.

We further probed this persistence phenomenon by examining levels of SARS-CoV-2 gRNA and nucleocapsid protein (N) in infected cells treated with nirmatrelvir or remdesivir. Because nirmatrelvir treatment in the clinical setting starts postinfection, we modified the in vitro experiment to mimic this scenario. Huh7-ACE2 cells were infected with SCoV-2/mNG at 0.5 MOI for 6 hours before the cells were washed and supplemented with nirmatrelvir or remdesivir at concentrations of 10 × IC_99_. After 24, 48, and 72 hours of treatment with drugs, samples of cells were washed and subjected to end point infectivity titration, as well as fixed for simultaneous imaging of viral gRNA by single-molecule RNA FISH [19] and N by immunofluorescence.

**Figure 3.**
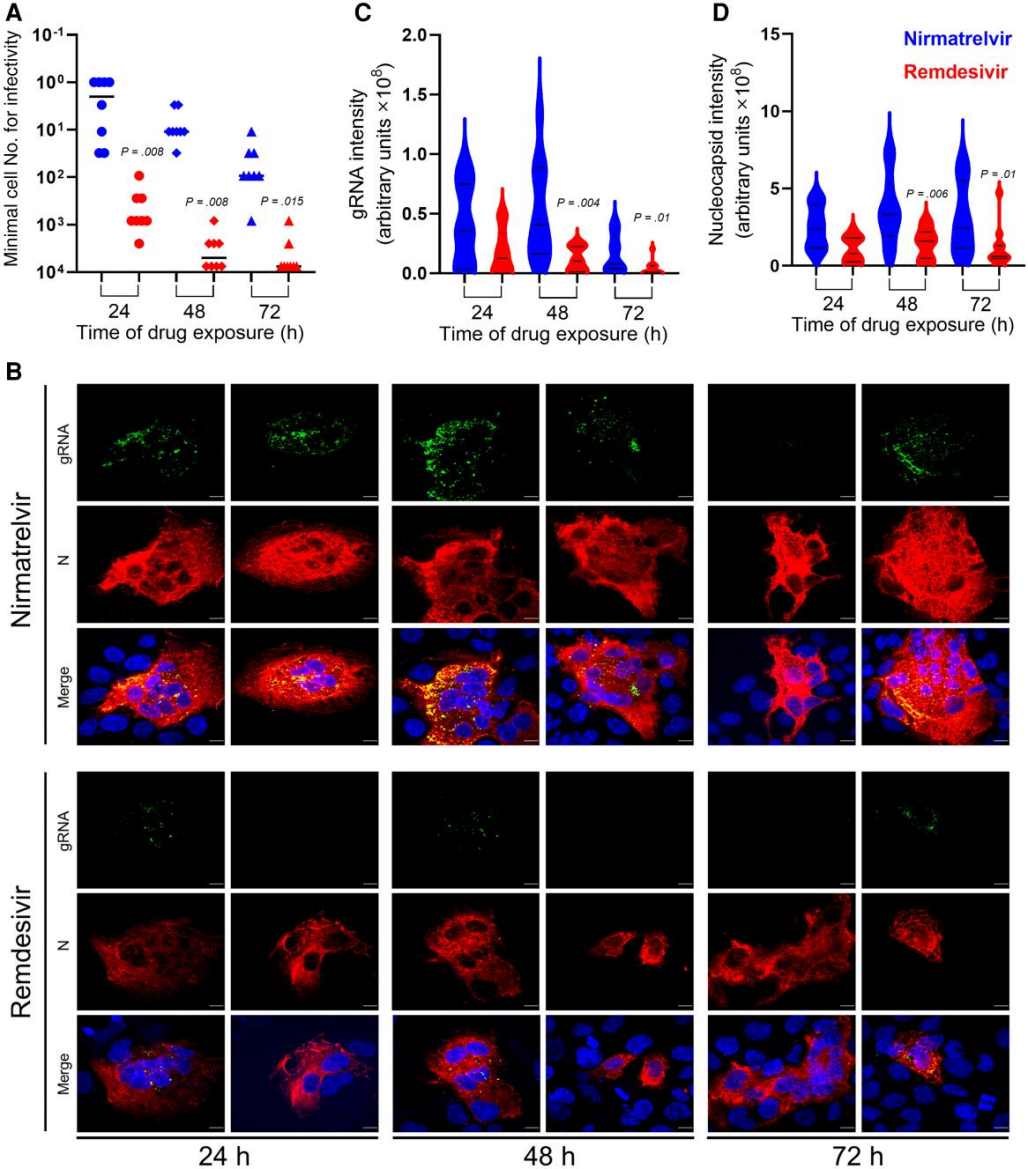
Persistence of infectious SARS-CoV-2 as well as gRNA and N in Huh7-ACE2 cells after drug treatment. Huh7-ACE2 cells were infected with SARS-CoV-2 at 0.5 MOI for 6 hours after which the virus was removed and replaced with growth media supplemented with either 20 µM nirmatrelvir or 1 µM remdesivir. *A*, Infectivity decay after removal of nirmatrelvir or remdesivir measured by minimal cell number read out similar to Figure 1. *B*, Two representative images from the same experiment demonstrate the persistence of gRNA (green) and viral N (red) at 24, 48, and 72 hours after nirmatrelvir or remdesivir treatment. Cell nuclei were stained with DAPI. Scale bar = 10 µm. *C* and *D*, Violin plots of the signal intensities for intracellular viral gRNA (*C*) and N (*D*) in infected cells (from Supplementary Figure 3). The exact *P* values for significant differences observed between nirmatrelvir and remdesivir are shown. Huh7-ACE2 cell culturing and experimentation in (*A*) were run in parallel tissue culture plates to those in (*B*–*D*), and images were collected from pure Huh7-ACE2 cell populations. Abbreviations: DAPI, 4′,6-diamidino-2-phenylindole; gRNA, genomic RNA; MOI, multiplicity of infection; N, nucleocapsid; SARS-CoV-2, severe acute respiratory syndrome coronavirus 2.

Cells harboring infectious virions were again detected at all time points from 24 to 72 hours after nirmatrelvir treatment (Figure 3*A*). Although, the observed infectious titers were higher than when an equivalent dose of nirmatrelvir was administered within 10 minutes of virus inoculation (Figure 1*B*), the infectivity decay half-lives were similar (24.6 hours vs 26.8 hours). The number of cells harboring infectious virus after remdesivir treatment was significantly lower at all time points (Figure 3*A*) by about 2 orders of magnitude. In the imaging studies, viral gRNA and N were visibly more abundant at all time points in cells treated with nirmatrelvir than those treated with remdesivir (Figure 3*B* and Supplementary Figure 3). This subjective observation was confirmed when the fluorescence signal intensities were quantified. Viral gRNA levels were indeed significantly higher at 48 and 72 hours after nirmatrelvir treatment compared to levels after remdesivir treatment (Figure 3*C*). Likewise, significantly higher levels of N were detected in nirmatrelvir-treated cells at 48 and 72 hours (Figure 3*D*). While these imaging results had similar trends to the infectivity decay data, their causal relationship is unknown. It should be noted that by allowing the virus to replicate for 6 hours prior to drugs treatment, syncytia formation could be detected after 24 hours posttreatment for all tested drugs (Figure 3*B*). While we cannot exclude the marginal influence of these multinuclear cells on our experimental set up prior to performing the end point dilution experiments (Figure 3*A*), our extensive wash step should eliminate as many multinuclear cells as possible, prior to counting the cells for end point titration. In addition, the percentage of syncytia in the cultures were similar across all treatments, suggesting that these fusion events occurred due to the production of viral proteins prior to exposure of the infected cell cultures to the drugs.

**Figure 4.**
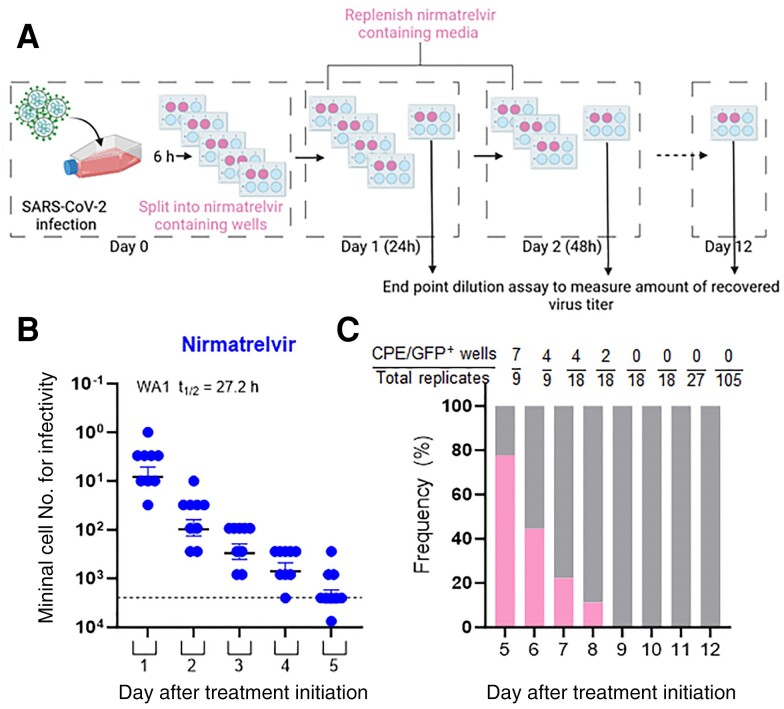
Persistence of infectious SARS-CoV-2, or lack thereof, through 12 days of nirmatrelvir treatment in vitro. *A*, Schematic layout of longer-term treatment of Huh7-ACE2 cells 6 hours postinfection with ic-SARS-CoV-2/WA1-mNG at MOI 0.5, followed by daily replenishment of media containing drug at 10 × IC_99_ and removal of batches of cells to measure the number of cells carrying infectious virus (created with BioRender.com). *B*, Infectivity decay for the first 5 days of nirmatrelvir treatment. Dotted lines indicate the upper limit of input cells (maximum cell number from which end point titration was performed at 3-fold dilutions) for the assay. *C*, Frequency of viral rebound after nirmatrelvir treatment from day 5 through day 12 of the experiment. Fractions above each bar show the number of replicates that yielded infectious virus (rebound positive). Abbreviations: CPE/GFP+, wells that showed infection by either cytopathic effect or mNeon fluorescence read-out; IC_99_, 99% inhibitory concentration; MOI, multiplicity of infection; SARS-CoV-2, severe acute respiratory syndrome coronavirus 2; t_1/2_, half-life.

Finally, we extended the course of nirmatrelvir treatment to 12 days to determine when viral rebound might be abolished in vitro (Figure 4*A*). During the course of the experiment, the cells were replenished with fresh medium containing drug every 24 hours, and batches of cells were taken out and subjected to limiting-dilution cultures to measure the number of cells carrying infectious SARS-CoV-2. Over the first 5 days, we noted similar rates of decay of infectious virus to those in the short-term experiments, with a half-life of 27.2 hours (Figure 4*B*). As the frequency of cells able to rebound and generate infectious viruses became lower, more replicates of limiting-dilution cultures were performed to increase the detection sensitivity. Infectious SARS-CoV-2 rebounds were detected through day 8 of the experiment, but none beyond that time point (Figure 4*C*).

## DISCUSSION

Our studies on SARS-CoV-2–infected cells in vitro suggest that there is an infectious form of the virus that persists intracellularly after nirmatrelvir or ensitrelvir treatment. We defined the rate of decay for this form using experimental half-life calculations to be approximately 1 day. Cell type-specific variations were measured by using more than a single human cell line, but irrespective of the cells used, the rates of decay were consistent. Rebound levels of infectious virus correlated to the levels of the gRNA and the nucleocapsid levels observed by RNA-FISH and immunofluorescence experiments. Assuming the drug and cellular pharmacokinetics in our study to be comparable to clinical disposition of protease inhibitors in humans, our data show that virus persistence outlives the 5 days of treatment where cells receive drug levels at or greater than the approved in vivo dose, reigniting SARS-CoV-2 infection as the drug is eliminated. This virologic hypothesis alone could explain Paxlovid rebound without implicating drug resistance, reinfection, or the immune system. This proposed explanation is also consistent with the observation that the viral rebound is more frequent in patients who were treated early [11] when the viral load is highest [15]. We hypothesize that either incoming viral ribonucleoprotein or replicating forms of the virus that were present in the cells prior to treatment with the protease inhibitor nirmatrelvir, can persist for a long period of time even in the presence of the drug, and can reinitiate infection once the drug is removed. Additional molecular and cellular studies are necessary to define, more precisely, the nature of these viral intermediates. Nevertheless, if our in vitro findings are reflective of the in vivo situation in patients, extending the course of nirmatrelvir treatment to 8–10 days should lower the probability of a viral rebound by 8 to 32-fold. Importantly, our data suggest that viral rebound could be abolished by extending the treatment period to longer than 5 days.

Viral persistence, which occurs postinfection and results in the lack of virus clearance in specific cells or tissues, is a common characteristic for many viruses from diverse viral families. For humans, the ability to establish persistent infection have been demonstrated for lentiviruses (human immunodeficiency virus 1 [HIV-1], human T-cell leukemia virus type 1 [HTLV]), herpes viruses (herpes simplex virus 1/2 [HSV1/2], Epstein-Barr virus [EBV], cytomegalovirus [CMV], varicella-zoster virus [VZV], human herpes virus 6/7 [HHV-6/7]), paramyxoviruses (measles), papovaviruses (human papillomavirus [HPV]), hepatitis B, C, and D viruses, and adenoviruses [24]. However, despite active ongoing research to elucidate the exact mechanisms that allow the initiation of a persistent infection with these viruses, our understanding of the tactics that they use to establish persistence and reverse latency in chronic infections is incomplete. We expect that the mechanism behind SARS-CoV-2 persistence mediated by protease inhibitors would require manifold approaches similar to such studies.

Studies unraveling the persistence of SARS-CoV-2 have been expanding over the last 2 years in immunocompromised and healthy individuals, with a recent study in a cohort of 381 individuals showing virus persistence for a period of 60 days or longer [25], with over 30% of these cases having a rebound measured by reverse transcription polymerase chain reaction (RT-PCR) and implicating this population as a major reservoir/carrier of the virus. While this study had a larger proportion of unvaccinated people, other studies have shown that the rebound phenomenon in healthy vaccinated and boosted individuals is likely to be relatively even higher due to longer clearance times despite lower peak values of infectious virus [15]. In fact, like other chronic viral infections, persistence has been a significant aspect of SARS-CoV-2 pathogenesis, especially in high-risk individuals in whom molecular correlates of diagnosis perform poorly [26–28]. Recent cross-sectional analyses in individuals who recovered from mild COVID-19 show a definitive association between virus persistence in multiple tissues and long COVID symptoms [29]. Replication of SARS-CoV-2 in nonmucosal sites impervious to regular detection (eg, swabs) is poorly studied and may further complicate the understanding of complete clearance of the virus from within the host. Our data from the long-term in vitro experiment using nirmatrelvir alone (Figure 4*C*) showed complete clearance of the virus only by day 8 of continued treatment, suggesting that extending the treatment with these 3CL protease inhibitors will pave the way for eliminating viral persistence. In fact, recent evidence in infected mice having an intact innate immunity and B cells [30] show that they recovered even with a late onset of treatment with Paxlovid or molnupiravir, but rebounded with measurable viral load in their upper respiratory tract 7 days after the completion of Paxlovid treatment, thereby providing further support for our proposal to prolong treatment to 8 days or longer.

Overall, our findings on the persistence of SARS-CoV-2 during treatment with nirmatrelvir is significant for reevaluating ongoing therapy and we advocate that the implications of prolonged treatment highlighted in our study must be clinically evaluated with a degree of urgency.

## Supplementary Data

Supplementary materials are available at *The Journal of Infectious Diseases* online (http://jid.oxfordjournals.org/). Supplementary materials consist of data provided by the author that are published to benefit the reader. The posted materials are not copyedited. The contents of all supplementary data are the sole responsibility of the authors. Questions or messages regarding errors should be addressed to the author.

## Supplementary Material

jiae385_Supplementary_Data

## Notes

***Author contributions***. D. D. H., Y. H., and Y. S. conceptualized the study. M. S. N. and Y. H. performed the virological and dose-response experiments. M. I. L. and Y. S. performed the smRNA-FISH and microscopy experiments. M. S. N., Y. H., Y. S., and D. D. H. wrote the manuscript.

***Financial support***. This work was supported by Andrew and Peggy Cherng; Samuel Yin; Barbara Picower and the JPB Foundation; Roger and David Wu; and the Bill and Melinda Gates Foundation (grant number PG012618). D. D. H. and Y. S. are supported by the National Institute of Allergy and Infectious Diseases (grant number 1U19AI1711401).
