# Supplementary material for: Persistence of an Infectious Form of SARS-CoV-2 After Protease Inhibitor Treatment of Permissive Cells In Vitro
Source: J Infect Dis. 2024 Aug 12;231(1):e68–76. doi: 10.1093/infdis/jiae385 (PMC11793057; doi:10.1093/infdis/jiae385)
Supplement: jiae385_Supplementary_Data [file jiae385_supplementary_data.docx]

**Supplementary Information**

**Supplementary Methods**

**Cell lines & virus strains**

Huh7 (human hepatocellular carcinoma) cells overexpressing human ACE2 cells were prepared by transducing Huh7 cells with lentivirus encoding human ACE2 and selecting for stable expression with 5 µg/mL blasticidin [1] and cultured in Essential Minimal Eagle’s medium (EMEM) containing 10% fetal calf serum (FCS) prior to use in dose–response and infectivity assays. Human lung tissue derived cells, line A549 obtained from ATCC (CCL-185) were engineered to overexpress the human angiotensin converting enzyme (ACE2) by stable transfection of a hu-ACE2 expressing lentiviral construct under puromycin selection [2] and cultured in EMEM with 10% FCS and obtained from BEI Resources (NR-53821). Cercopithecus aethiops kidney epithelial cells expressing transmembrane protease serine 2 and human ACE2 (Vero-TMPRSS2) were obtained from BEI Resources (NR-54970). An infectious clone of SARS-CoV-2 WA1 containing the mNeonGreen reporter (ic-SARS-CoV-2/WA1-mNG) was obtained from the World Reference Center of Emerging Viruses and Arboviruses (WRCEVA) at the University of Texas Medical Branch [3] and propagated using Vero E6 cells at the high containment laboratory at Aaron Diamond AIDS Center at Columbia University Medical Center. The resultant progeny virions were titered in Vero E6 cells prior to use in dose-response and infectivity experiments. Omicron BA.1.1 isolate hCoV-19/USA/HI-CDC-4359259-001/2021 was procured from BEI Resources (NR-56475), passaged in Vero-TMPRSS2 cells and titered using Reed & Muench method prior to performing infectivity experiments in these cells.

**Dose-response inhibition of virus**

Triplicates of serial (1 in 4) dilution for each drug starting at 100 µM concentration was made in EMEM (+10% FCS) and plated on the surface of a monolayer of 20,000 target cells per well seeded overnight, at 100 µL volumes in a 96-well plate format. SARS-CoV-2 was added 10 to 15 min later at 0.5 MOI per well. Cells were incubated at 37^o^C/5%CO_2_ for 70h prior to measuring cytopathic effects (CPE) under the microscope. CPE was determined relative to the wells lacking any treatment (virus controls) and plotted as a function of the dose using non-linear asymmetric five-parameter dose-response curve in GraphPad Prism version 9.4 to determine the corresponding 50% and 99% inhibitory concentrations of the drug in the cell line. Dose-response curves were performed at least three times in each cell line to determine the IC_99_ values of all the drugs. Since efflux pumps like P-glycoprotein did not affect our dose response curves in multiple rounds of testing inhibition against protease inhibitors [4], probably due to absence or dysfunctionality of the pumps in these cells [5, 6], we measured the dose-response in all cells in the absence of inhibitors of such pumps.

**Determination of drug cytotoxicity in tested cells**

Nirmatrelvir, Ensitrelvir, GC-376 and Remdesivir stocks in DMSO were serially diluted with 5-fold dilutions into EMEM (+10% FCS) and overlaid on overnight grown monolayer of 20,000 cells per well of Huh7-ACE2, A549-ACE2 and Vero-TMPRSS2 cells, 100 µL volumes in a 96-well plate format. Cells were incubated at 37^o^C/5%CO2 for 48h before treatment with a proprietary water-soluble dye that is reduced by live cells (Colorimetric Cell cytotoxicity kit; Abcam ab112118). Imatinib, a cell cycle arresting tyrosine kinase inhibitor was used as a positive control for cytotoxicity in the cells. Each drug was tested in three replicates per cell line following instructions provided by the manufacturer and values were plotted on GraphPad Prism v10 as percent cell viability at 48h over the range of concentrations per drug.

**Determination of minimal cell number in long term post-infection study with nirmatrelvir**

The schematic of the long-term treatment with nirmatrelvir in Huh7-ACE2 cells are provided in schematic in **Figure 4A**. Briefly, at the end of infection, the cells were trypsinized and washed with EMEM containing 3% fetal calf serum prior to seeding 6-well plates with a low seeding number of cells that would allow for proliferation of the cells for a longer period in the wells. Cells were immediately treated with medium containing nirmatrelvir at 10-fold higher than its 99% inhibitory dose in the cells. Incubation of the cells occurred at 37C/5%CO_2_ till the end of the experiment. Fresh daily stocks of drugs were prepared in EMEM at the same concentration of the drug, and this was used to replenish the wells with fresh drug every 24h. The concentration of the fetal calf serum was reduced to 5% for days 5-9 and to 3% for days 10-12 in the assay to allow maintenance of the culture for the extended period of in vitro study.

**Single molecule RNA FISH and immunofluorescence**

Briefly, sterile 13mm round glass coverslips were placed in a 12-well plate and 75,000 Huh7-ACE2 cells were plated in 1ml DMEM growth media per well, 24 h prior for infection and drugs treatment. At every time point, the cells were washed 3 times with 1x PBS and fixed using 4% formaldehyde in PBS (10 min) followed by additional 3 washes with 3 times with 1x PBS. Samples that were taken at the 24 and 48 h time points were stored at 4^o^C and processed together with the 72 h samples. For nucleocapsid detection at the immunofluorescence step, mouse monoclonal anti- SARS-CoV-1/2 N Antibody (clone 1C7C7; Sigma Cat# ZMS1075) at a concentration of 1µg/ml was used as the primary antibody, and Alexa Fluor 647 goat anti-mouse (ThermoFisher Scientific Cat# A21236) at a concentration of 2µg/ml was used as the secondary antibody. For single-molecule RNA FISH to detect the positive strand of the SARS-CoV-2 genome, probe sequences were designed using Stellaris Probe Designer version 4.2 (<https://www.biosearchtech.com/stellaris-designer>) with the following parameters: organism, human; masking level, 5; oligo length, 20 nt; minimum spacing length, 3 nt and the nucleotide sequence of ORF1a from the ic-SARS-CoV-2/WA1-mNG SARS-CoV-2 [3] similarly to previous published study [7]. Oligonucleotide probes were purchased labeled with TAMRA dye and used at a 250nM final concentration.

**Quantitation & Statistical Analysis**

**Linear regression analysis to measure decay half-life of infectious virus**

The minimal cell numbers from all the replicates that are required to infect Vero-TMPRSS2 indicator cells at each time point were used to generate a linear regression curve for each drug in each experiment. The reciprocal of the cell number for each of the 9 replicates for every drug was averaged to determine the average frequency of infectivity per time point per drug. The slope of the decay of the average frequency was calculated using a simple linear regression analysis in GraphPad Prism version 10.0 to determine the half-life (t_1/2_) of the infectious material when treated with that drug at a particular dose. A comparison of the half-life of each drug at every dose was calculated and tabulated. Only those points that were quantifiable (≥LOQ of assay) were used to calculate the slope and the corresponding half-lives were represented as less than the obtained number.

**Statistical analysis for measuring significance between groups**

Statistics for significance between test groups in each experiment when shown were performed using the one-tailed student’s t-test in GraphPad Prism.

**References in Supplementary Methods:**

1. Liu H, Iketani S, Zask A, et al. Development of optimized drug-like small molecule inhibitors of the SARS-CoV-2 3CL protease for treatment of COVID-19. Nat Commun **2022**; 13(1): 1891.

2. Ikhlas S, Usman A, Kim D, Cai D. Exosomes/microvesicles target SARS-CoV-2 via innate and RNA-induced immunity with PIWI-piRNA system. Life Sci Alliance **2022**; 5(3): e202101240.

3. Xie X, Muruato A, Lokugamage KG, et al. An Infectious cDNA Clone of SARS-CoV-2. Cell Host Microbe **2020**; 27(5): 841-848.

4. Iketani S, Mohri H, Culbertson B, et al. Multiple pathways for SARS-CoV-2 resistance to nirmatrelvir. Nature **2023**; 613(7944): 558-64.

5. Jouan E, Le Vee M, Denizot C, Parmentier Y, Fardel O. Drug Transporter Expression and Activity in Human Hepatoma HuH-7 Cells. Pharmaceutics **2016**; 9(1): 3.

6. Vries Md, Mohamed AS, Prescott RA, et al. A Comparative Analysis of SARS-CoV-2 Antivirals Characterizes 3CLpro Inhibitor PF-00835231 as a Potential New Treatment for COVID-19. J Virol **2021**; 95(7):e01819-20.

7. Lee JY, Wing PAC, Gala DS, et al. Absolute quantitation of individual SARS-CoV-2 RNA molecules provides a new paradigm for infection dynamics and variant differences. Elife **2022**; 11.

**

**

**Supplementary Figure 1: Dose-response inhibition of SARS-CoV-2 by protease and polymerase inhibitors.** Dose titration of drugs in (A) Huh7-ACE2 cells infected with WA1, and (B) A549-ACE2 cells infected with WA1 virus. All viruses were used at MOI 0.5 to obtain the IC50 and IC99 of inhibition of the proteases and polymerase inhibitor. DMSO (vehicle) was serially diluted into medium and used as negative control (black line). (C) Table showing potency of each drug in respective cell lines. (D) Dose titration of drugs in Huh7-ACE2 cells, A549-ACE2 cells and Vero-TMPRSS2 cells showing toxicity of indicated drug doses on cells at 48h post incubation. The dotted black line in each graph indicates 50% value while the dotted grey line when shown indicates 99% value.

**
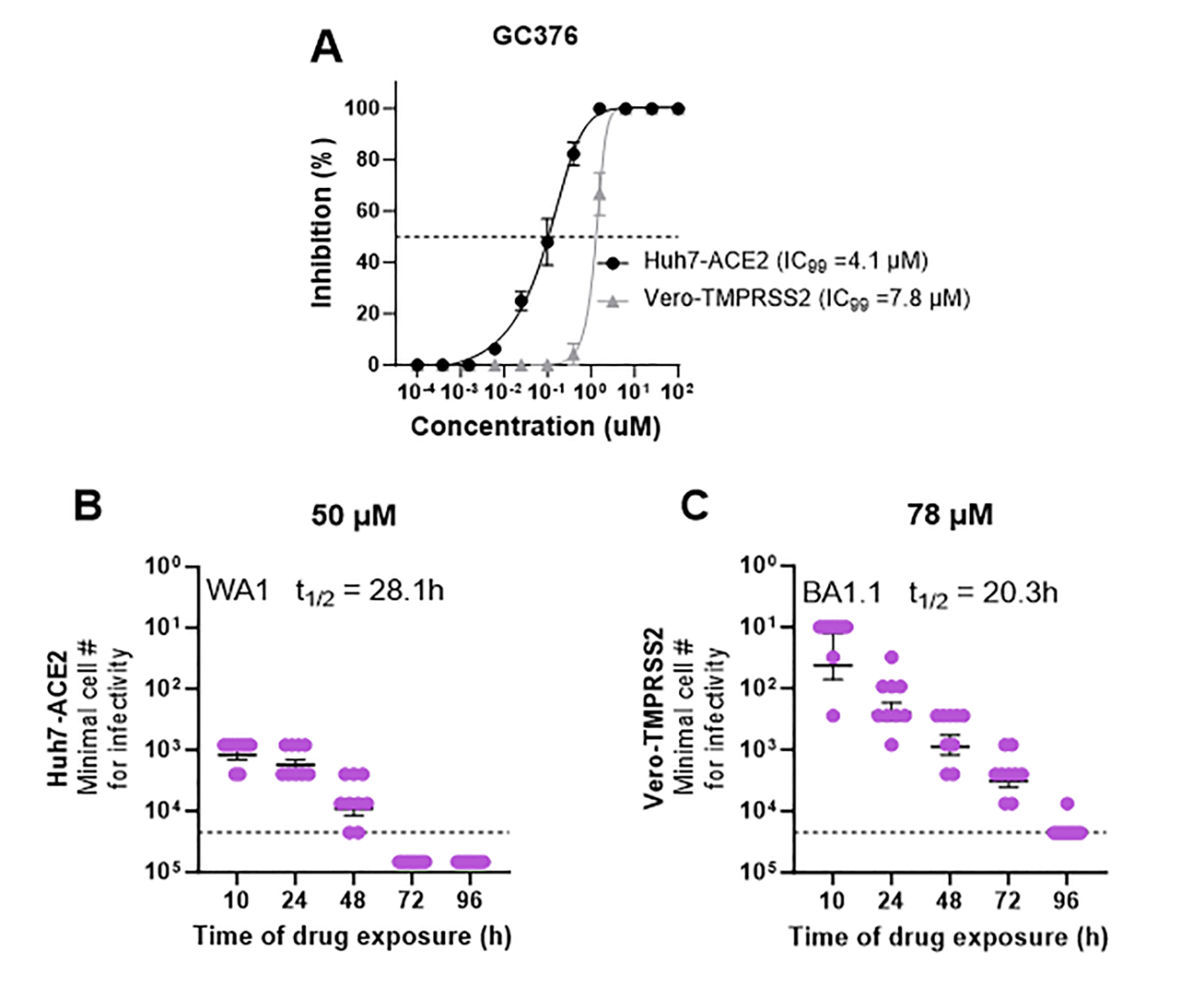
**

**Supplementary Figure 2: Testing for SARS-CoV-2 persistence during concurrent treatment with another protease inhibitor (GC-376).** A) Dose-response curves for GC-376 were measured to establish the IC_99_ values of the drug against ic-SARS-CoV-2/WA1-mNG in Huh7-ACE2 cells and against Omicron BA.1.1 in Vero-TMPRSS2 cells. B) Endpoint titers for Huh7-ACE2 cells treated with 50 µM GC-376 (10X IC_99_) at each time point of drug withdrawal. Half-life calculated using linear regression from data points that are quantifiable (≥LOQ of assay) shown in the inset text. C) Endpoint titers for Vero-TMPRSS2 cells treated with 78 µM GC-376 (10X IC_99_) at each time point of drug withdrawal. Half-life calculated using linear regression shown in the inset text. Note: A549-ACE2 cells resulted in no virus suppression at 10-fold dosing once drug is withdrawn likely due to weak inhibitory activity of the drug in the cell with higher IC_99_ values (13.4 µM). Therefore, it is not suitable for endpoint titration.

**
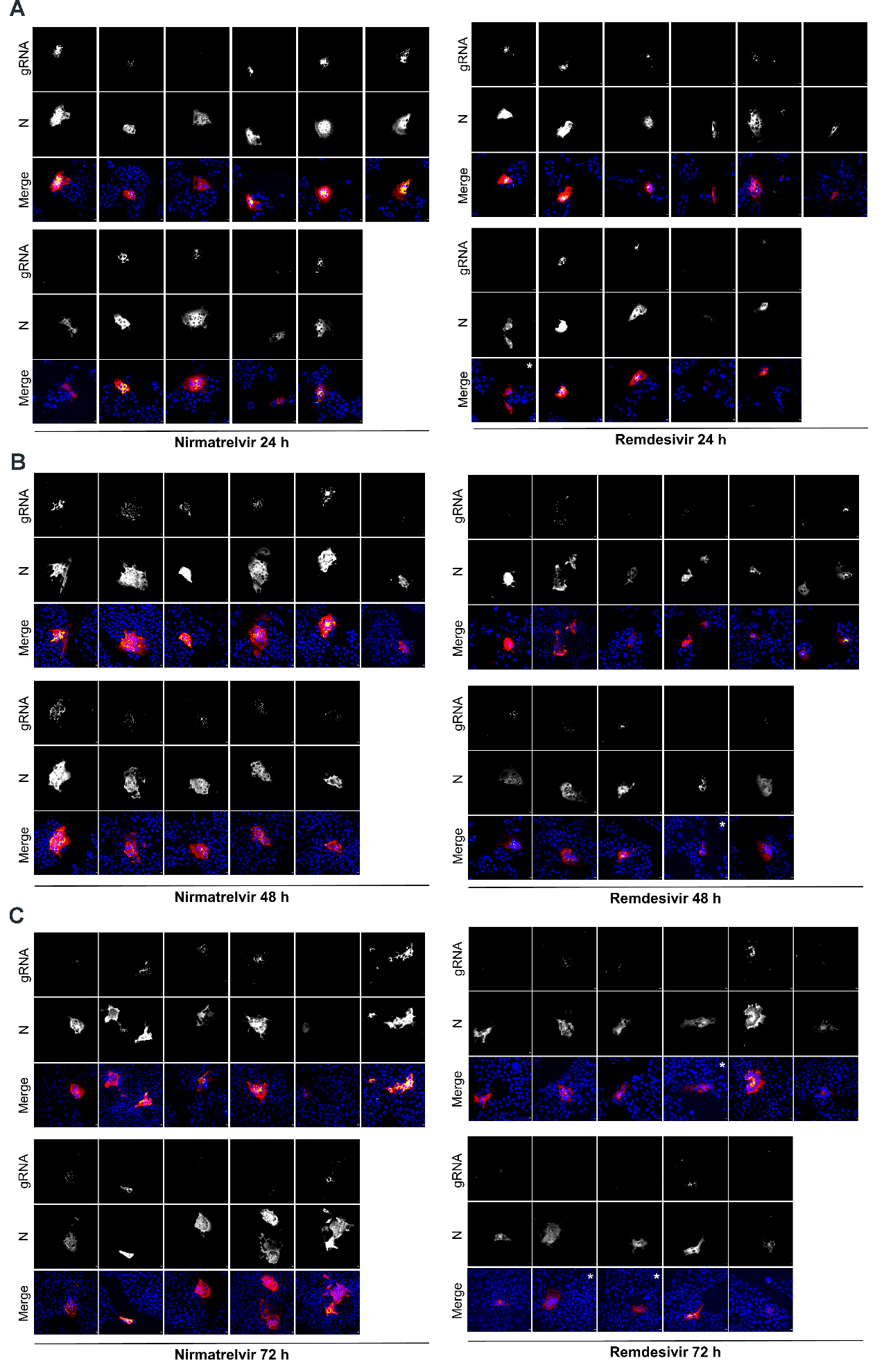
**

**Supplementary Figure 3: Simultaneous detection of SARS-CoV-2 genomic RNA and nucleocapsid protein in Huh-7 infected cells post nirmatrelvir or remdesivir treatment.** Huh7-ACE2 were infected with SARS-CoV-2 at 0.5 MOI for 6 hours after which the virus was removed and replaced with growth media supplemented with either 20 µM nirmatrelvir or 1µM remdesivir. At 24 hours (A), 48 hours (B) or 72 hours (C), cells were fixed and processed to detect the genomic viral RNA (green) as well as the viral nucleocapsid protein (red) using RNA-FISH with specific probes together with immunoflurecsence using anti-SARS-CoV-2 nucleocapsid antibodies. Cell nucleus was stained with DAPI (blue). White star indicates higher exposure setting in the red channel to detect weak nucleocapsid signal. For each time point and condition, 11 random fields from the same experiment are shown. Scale bar = 10µm.
